# Supplementary material for: Treating Parkinson’s Disease with Human Bone Marrow Mesenchymal Stem Cell Secretome: A Translational Investigation Using Human Brain Organoids and Different Routes of In Vivo Administration
Source: Cells. 2023 Nov 2;12(21):2565. doi: 10.3390/cells12212565 (PMC10650433; doi:10.3390/cells12212565)
Supplement: Supplementary file 1 [file cells-12-02565-s001.zip › cells-2609980-supplementary.pdf]

Supplementary Data

Mendes-Pinheiro et al.

Treating Parkinson's Disease with human Bone Marrow Mesenchymal Stem Cell Secretome: A translational investigation using human brain organoids and different routes of *in vivo* administration.

Bárbara Mendes-Pinheiro[1,2,ǂ], Jonas Campos[1,2,ǂ], Ana Marote[1,2], Carina Soares-Cunha[1,2], Sarah L. Nickels[3], Anna S. Monzel[3], Jorge. R Cibrão[1,2], Eduardo Loureiro-Campos[1,2], Sofia C. Serra[1,2], Sandra Barata-Antunes[1,2], Sara Duarte-Silva[1,2], Luísa Pinto[1,2], Jens C. Schwamborn[3*], António J. Salgado[1,2*#]

ǂThese authors are equally contributors.

*These authors share senior co-authorship.

[1] Life and Health Sciences Research Institute (ICVS), School of Medicine, University of Minho, Campus de Gualtar, 4710-057 Braga, Portugal

[2] ICVS/3B's – PT Government Associate Laboratory, 4805-017Guimarães, Portugal

[3] Luxembourg Centre for Systems and Biomedicine (LCSB), University of Luxembourg, L-4367 Belvaux, Luxembourg.

#Corresponding author: A. J. Salgado, PhD, Life and Health Sciences Research Institute (ICVS), School of Medicine, University of Minho, Campus de Gualtar, 4710-057 Braga, Portugal. E-mail: asalgado@med.uminho.pt; Tel: +351 253 60 49 47; Fax: +351 253 60 48 20

*These authors share senior co-authorship.

In order of appearance in the text

a.

WT Name	Age of sampling	Sex	References	Database Number
K7	81	F	iPSCs Reinhardt et al., 2013	200
T12	53	F	iPSCs Reinhardt et al., 2013	232
COR	55	M	iPSCs Coriell GM23338	225

b.

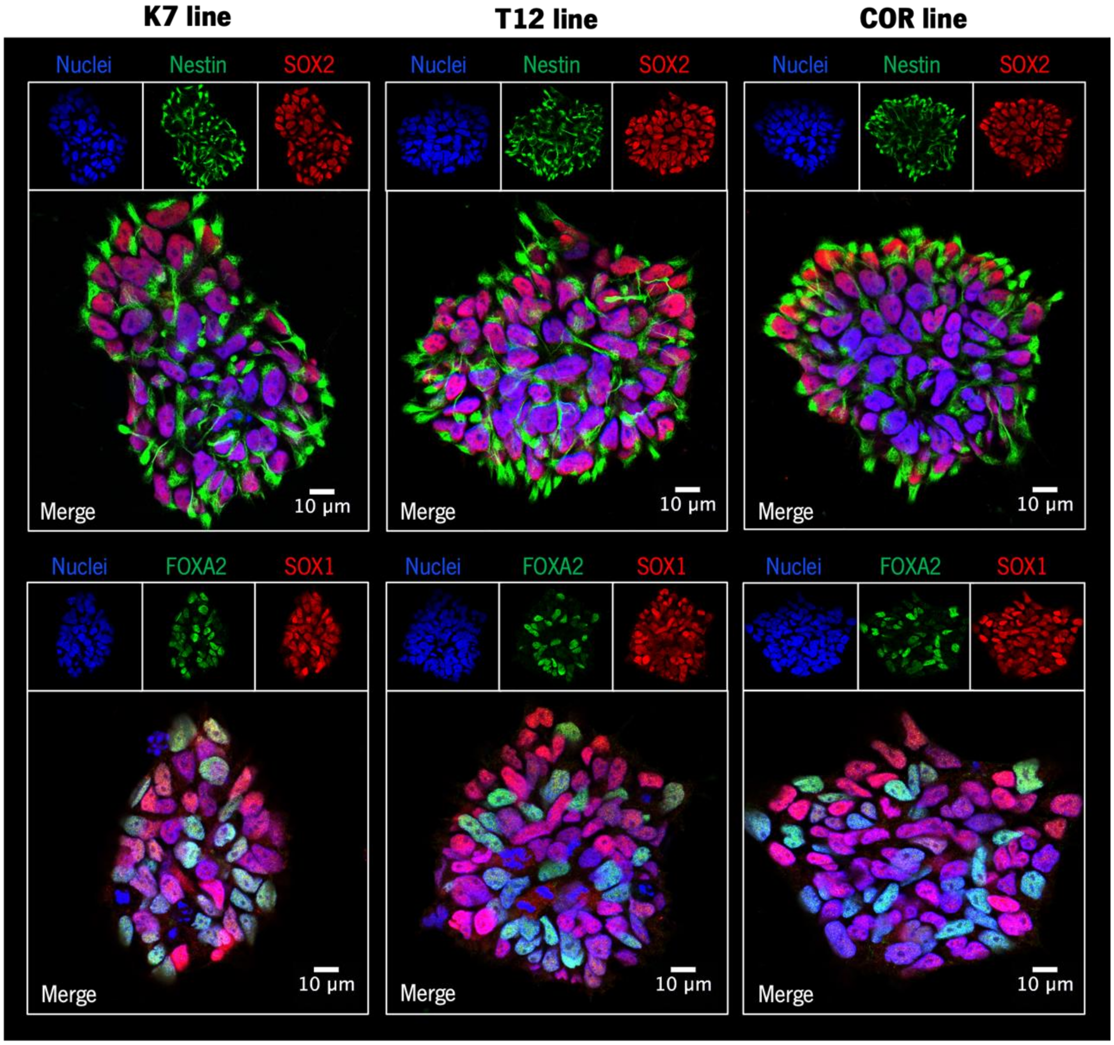

Figure S1. Cell lines used in this study to generate hMOs (passage 14). a) Donor characteristics of cell lines[1]. b) representative images of hvNESC morphology and expression of neuronal progenitor (Nestin, SOX1, and SOX2) and midbrain floorplate markers (FOXA2).

Table S1. List of primary antibodies.

Antibody	Specie	Dilution	Source	Reference
Dopamine	rabbit	1:500	ImmuSmol	IS1005
EN1	goat	1:300	Santa Cruz	sc-46101
FOXA2	mouse	1:250	Santa Cruz	sc-101060
GFAP	chicken	1:500	Millipore	AB5541
GIRK2	goat	1:200	Abcam	ab65096
Nestin	mouse	1:100	Millipore	MAB5326
PAX6	rabbit	1:300	Covance	PRB-278P
SOX1	goat	1:100	R&D systems	AF3369
SOX2	goat	1:100	R&D systems	AF2018
S100β	mouse	1:100	Sigma	S2532
TH	chicken	1:1000	Abcam	ab76442

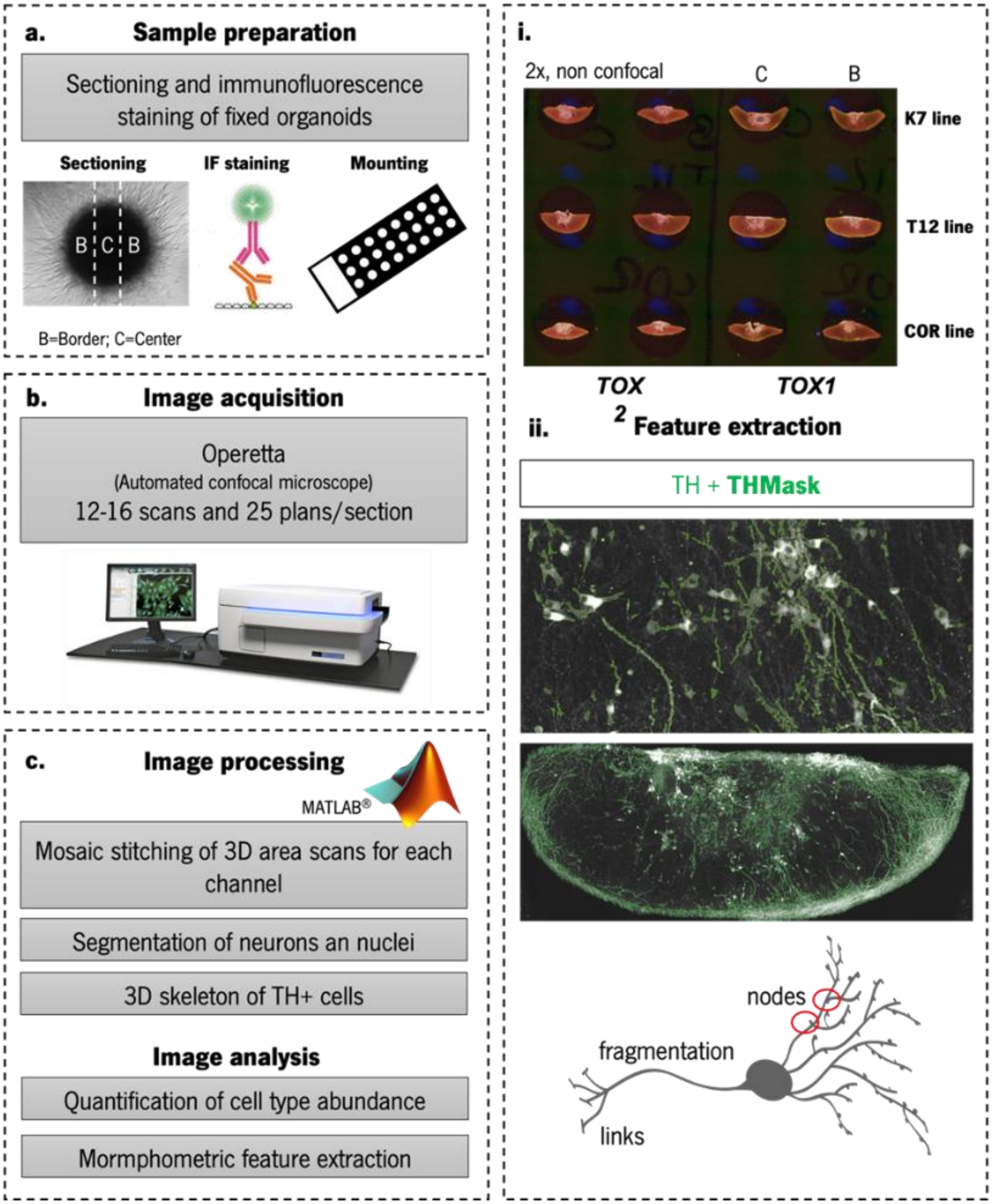

Figure S2. High-content image analysis workflow. a) sample preparation: organoids were sectioned and prior to immunofluorescence staining separated into border and center sections. Organoid sections were mounted on an object slide containing a grid for automated image acquisition; b) image acquisition: 12-16 area scans in 25 planes were acquired using an automated confocal microscope (operetta); c) images were exported in MATLAB and area scans were stitched. On the obtained 3D image, masks were generated for dopaminergic neurons in order to quantify cell type abundance and morphometric features; i) 2x non confocal image representation of organoid slices; ii) feature extraction:

Dopaminergic neuronal complexity was quantified by extracting cellular features such as TH mask, neurite nodes, links, and fragmentation. Adapted from (Monzel et al., 2020) [3].

Feature	Description
Nuclei mask	Count of nuclear positive pixels
Nuclei dead mask	Count of nuclear dead pixels
Nuclei dead/nuclei	Count of nuclear dead pixels / Hoechst
TUJ1 mask	Count of TUJ1 mask pixels
TUJ1+/Nuclei	Sum of TUJ1+ pixels / Hoechst
MAP2 mask	Count of MAP2 mask pixels
MAP2+/Nuclei	Sum of MAP2+ pixels / Hoechst
TH mask	Count of TH mask pixels
TH+/Nuclei	Sum of TH+ pixels / Hoechst
TH percentage	Percentage of TH+ cells in the perinuclear zone of segmented nuclei
TH skeleton	Count of TH skeleton pixel (red). Skeleton is calculated using a thinning function resulting in a simplified representation of the neuronal branching. The skeleton allows identifying nodes and links 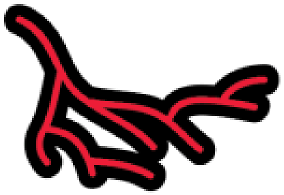
Nodes	Total number of points located at a bifurcation of neuronal branching (red points), generated from the TH skeleton 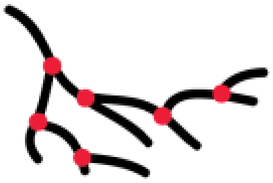
Links	Total number of connecting segments originated from the nodes (red lines), generated from the TH skeleton 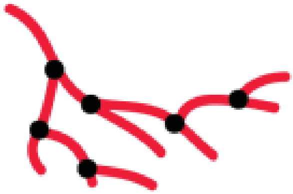

TH fragmentation	Surface to volume ratio of TH mask

Table S2. Features from image analysis (adapted from (Smits et al., 2019))[2]**.**

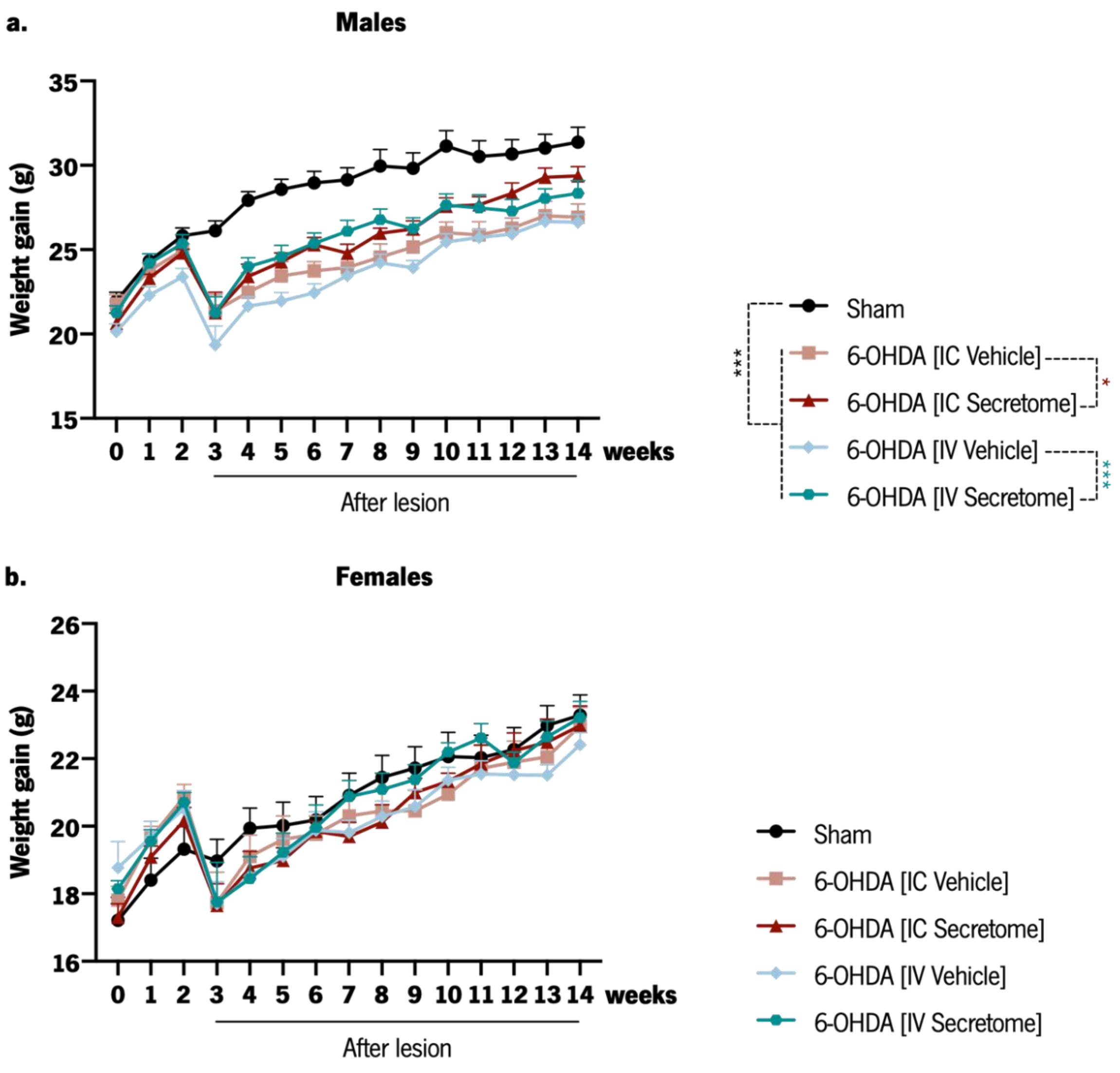

Figure S3. Weight gain throughout the study. Significant differences were found in the weight gain in males after lesion (Sham vs 6-OHDA-lesioned animals), and after treatments; b) No significant differences were found in the weight gain for females. These results did not interfere with the motor performance of the animals on the different tasks, as observed

by using the bodyweight as co-variable or splitting the data by sex (data not shown). n[males] = 6-9/ n[females] = 6-8 for each group used. Statistical summary in Table S1. Data are presented as mean ± SEM. *$p < 0.05$, ***$p < 0.001$.

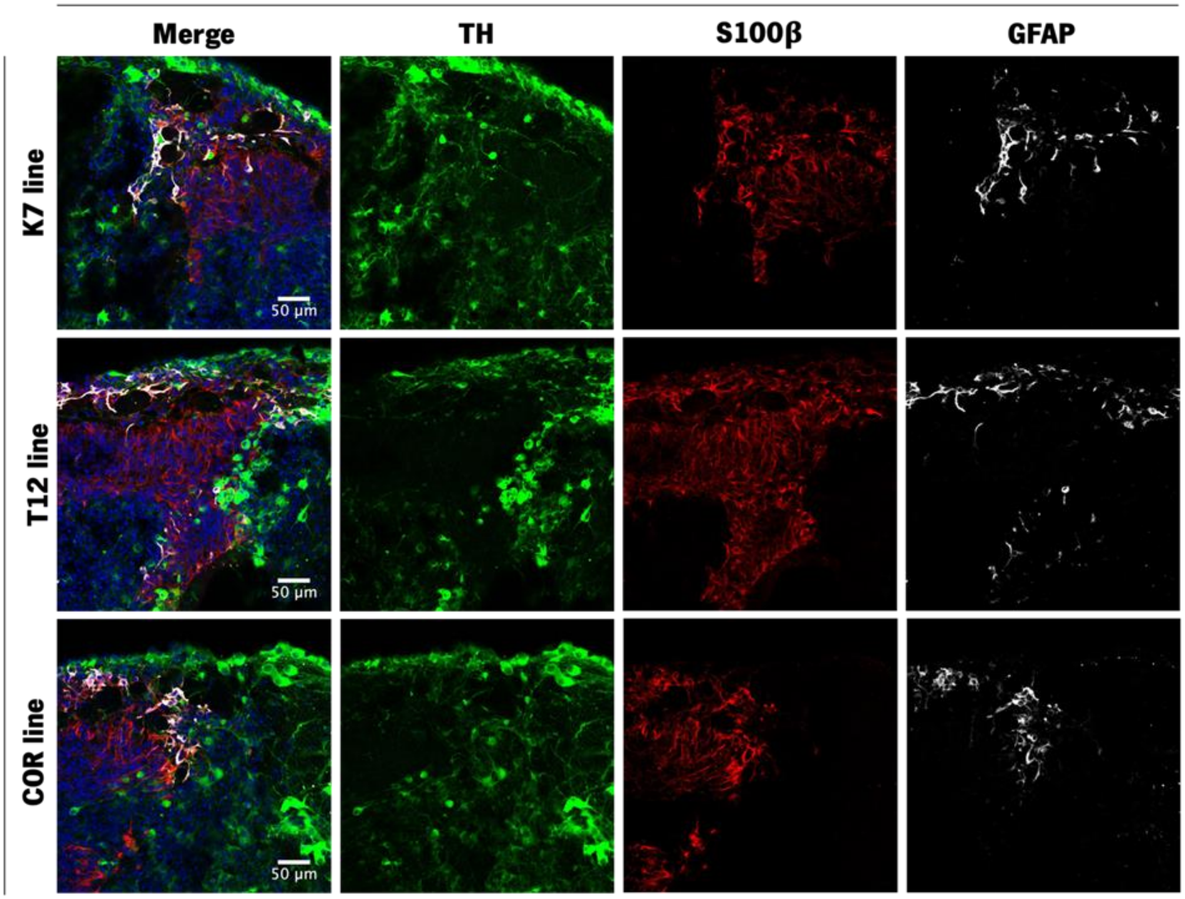

Figure S4. Characterization of glial differentiation at day 30 of the differentiation protocol. Immunofluorescence staining for astrocytic markers S100β (red) and GFAP (white).

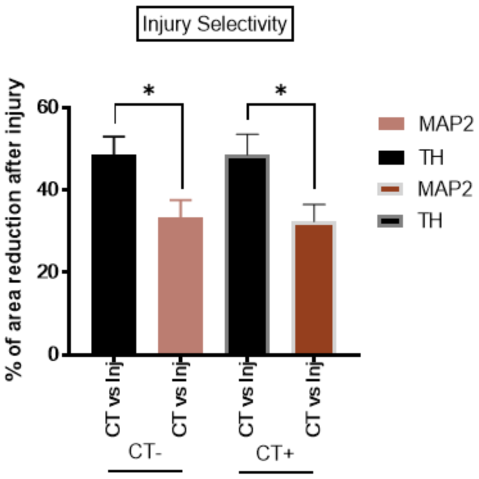

Figure S5. Selectivity of 6-OHDA-induced degeneration in neuronal populations. Measurements of area marker (TH or MAP2) reduction after injury demonstrate a higher degree of degeneration of the dopaminergic cell population. Data are presented as mean ± SEM. n= 24. Unpaired T-test; CT- (TH vs MAP2 Cohen's d=0.774); CT+ (TH vs MAP2 Cohen's d=0.718). *$p < 0.05$.

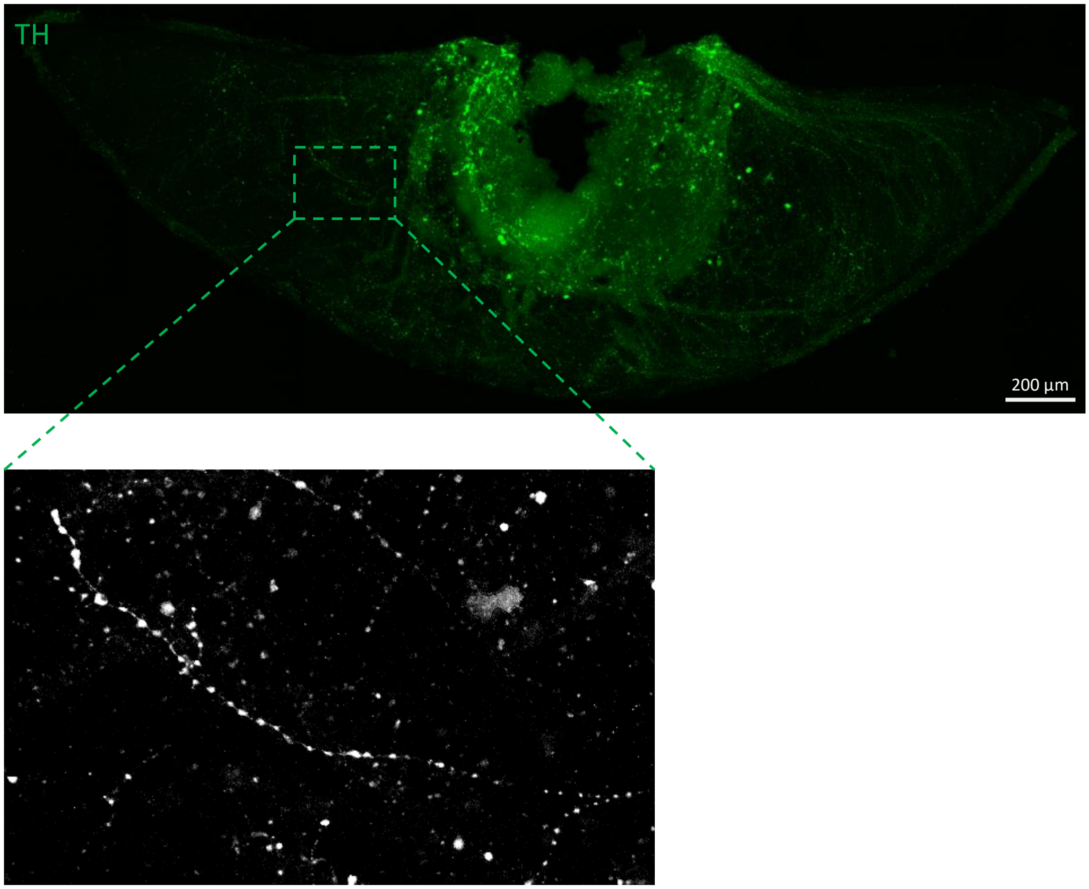

Figure S6. Example of a fragmented neurite after 6-OHDA treatment. It is possible to observe a clearly beaded and broken fiber marked with TH in the hMO exposed to the neurotoxin.

Measure	Statistical Report	***Effect size** (weeks after treatments)
**Rotameter test**<br>*Net-contralateral rotations*	U=0, $p < 0.001$	-
**Motor swimming test**<br>*Latency (s)*	Time: $F_{(2.67, 176.1)} = 90.72$, $p < 0.001$<br>Group: $F_{(4, 68)} = 10.91$, $p < 0.001$<br>Time x Group: $F_{(12, 199)} = 5.12$, $p < 0.001$	**1 week**<br>IC Vehicle vs IC Secretome: Cohen's d = 0.37<br>IV Vehicle vs IV Secretome: Cohen's d = 1.3<br>**4 weeks**<br>IC Vehicle vs IC Secretome: Cohen's d = 0.97<br>IV Vehicle vs IV Secretome: Cohen's d = 1.03<br>**7 week**<br>IC Vehicle vs IC Secretome: Cohen's d = 0.96<br>IV Vehicle vs IV Secretome: Cohen's d = 1.46
	**Multiple comparisons**<br>Sham vs 6-OHDA [IC Vehicle]: $p < 0.001$<br>Sham vs 6-OHDA [IC Secretome]: $p < 0.001$<br>Sham vs 6-OHDA [IV Vehicle]: $p < 0.001$<br>Sham vs 6-OHDA [IV Secretome]: $p < 0.001$<br>6-OHDA [IC Vehicle] vs 6-OHDA [IC Secretome]: $p = 0.180$<br>6-OHDA [IV Vehicle] vs 6-OHDA [IV Secretome]: $p = 0.002$<br>6-OHDA [IC Secretome] vs 6-OHDA [IV Secretome]: $p = 0.655$	

**Pole test**<br><br>*Time to reach the cage (s)*	Time: $F_{(3, 201)} = 19.57$, $p < 0.001$<br><br>Group: $F_{(4, 69)} = 9.92$, $p < 0.001$<br><br>Time x Group: $F_{(12, 201)} = 5.03$, $p < 0.001$<br><br>**Multiple comparisons**<br><br>Sham vs 6-OHDA [IC Vehicle]: p < 0.001<br><br>Sham vs 6-OHDA [IC Secretome]: p = 0.014<br><br>Sham vs 6-OHDA [IV Vehicle]: p < 0.001<br><br>Sham vs 6-OHDA [IV Secretome]: p = 0.047<br><br>6-OHDA [IC Vehicle] vs 6-OHDA [IC Secretome]: p = 0.378<br><br>6-OHDA [IV Vehicle] vs 6-OHDA [IV Secretome]: p = 0.025<br><br>6-OHDA [IC Secretome] vs 6-OHDA [IV Secretome]: p = 0.987	**1 week**<br><br>IC Vehicle vs IC Secretome: Cohen's d = 0.71<br><br>IV Vehicle vs IV Secretome: Cohen's d = 0.94<br><br>**4 weeks**<br><br>IC Vehicle vs IC Secretome: Cohen's d = 0.64<br><br>IV Vehicle vs IV Secretome: Cohen's d = 0.92<br><br>**7 week**<br><br>IC Vehicle vs IC Secretome: Cohen's d = 0.90<br><br>IV Vehicle vs IV Secretome: Cohen's d = 1.13
**Beam balance walk test**<br><br>*Latency (s)*	Time: $F_{(2.53, 159.1)} = 19.70$, $p < 0.001$<br><br>Group: $F_{(4, 70)} = 8.90$, $p < 0.001$<br><br>Time x Group: $F_{(12, 189)} = 2.25$, $p < 0.05$<br><br>**Multiple comparisons**<br><br>Sham vs 6-OHDA [IC Vehicle]: p < 0.001<br><br>Sham vs 6-OHDA [IC Secretome]: p < 0.001<br><br>Sham vs 6-OHDA [IV Vehicle]: p < 0.001<br><br>Sham vs 6-OHDA [IV Secretome]: p = 0.0034<br><br>6-OHDA [IC Vehicle] vs 6-OHDA [IC Secretome]: p = 0.681<br><br>6-OHDA [IV Vehicle] vs 6-OHDA [IV Secretome]: p = 0.008<br><br>6-OHDA [IC Secretome] vs 6-OHDA [IV Secretome]: p = 0.441	**1 week**<br><br>IC Vehicle vs IC Secretome: Cohen's d = 0.53<br><br>IV Vehicle vs IV Secretome: Cohen's d = 0.68<br><br>**4 weeks**<br><br>IC Vehicle vs IC Secretome: Cohen's d = 0.05<br><br>IV Vehicle vs IV Secretome: Cohen's d = 0.56<br><br>**7 week**<br><br>IC Vehicle vs IC Secretome: Cohen's d = 0.50<br><br>IV Vehicle vs IV Secretome: Cohen's d = 1.81
**TH+ labelling**<br><br>*Dorsal striatum*	Time: $F_{(4, 30)} = 49.71$, $p < 0.001$<br><br>**Multiple comparisons**<br><br>Sham vs 6-OHDA [IC Vehicle]: p < 0.001<br><br>Sham vs 6-OHDA [IC Secretome]: p < 0.001	IC Vehicle vs IC Secretome: Cohen's d = 2.9<br><br>IV Vehicle vs IV Secretome: Cohen's d = 1.5

	Sham vs 6-OHDA [IV Vehicle]: $p < 0.001$<br>Sham vs 6-OHDA [IV Secretome]: $p < 0.001$<br>6-OHDA [IC Vehicle] vs 6-OHDA [IC Secretome]: $p < 0.001$<br>6-OHDA [IV Vehicle] vs 6-OHDA [IV Secretome]: $p = 0.049$<br>6-OHDA [IC Secretome] vs 6-OHDA [IV Secretome]: $p = 0.026$	
**TH+ cells**<br>*SNpc*	Time: $F_{(4, 30)} = 60.92$, $p < 0.001$<br>**Multiple comparisons**<br>Sham vs 6-OHDA [IC Vehicle]: $p < 0.001$<br>Sham vs 6-OHDA [IC Secretome]: $p < 0.001$<br>Sham vs 6-OHDA [IV Vehicle]: $p < 0.001$<br>Sham vs 6-OHDA [IV Secretome]: $p < 0.001$<br>6-OHDA [IC Vehicle] vs 6-OHDA [IC Secretome]: $p = 0.005$<br>6-OHDA [IV Vehicle] vs 6-OHDA [IV Secretome]: $p = 0.370$<br>6-OHDA [IC Secretome] vs 6-OHDA [IV Secretome]: $p = 0.127$	IC Vehicle vs IC Secretome: Cohen's d = 1.7<br>IV Vehicle vs IV Secretome: Cohen's d = 1.17
**Weight gain**<br>*Males*	Time: $F_{(3.35, 111.9)} = 136.5$, $p < 0.001$<br>Group: $F_{(4, 34)} = 12.18$, $p < 0.001$<br>Time x Group: $F_{(56, 468)} = 3.14$, $p < 0.001$<br>**Multiple comparisons**<br>Sham vs 6-OHDA [IC Vehicle]: $p < 0.001$<br>Sham vs 6-OHDA [IC Secretome]: $p < 0.001$<br>Sham vs 6-OHDA [IV Vehicle]: $p < 0.001$<br>Sham vs 6-OHDA [IV Secretome]: $p < 0.001$<br>6-OHDA [IC Vehicle] vs 6-OHDA [IC Secretome]: $p = 0.03$<br>6-OHDA [IV Vehicle] vs 6-OHDA [IV Secretome]: $p < 0.001$<br>6-OHDA [IC Secretome] vs 6-OHDA [IV Secretome]: $p = 0.998$	-

**Weight gain**<br>*Females*	Time: $F_{(3.67, 113.6)} = 121.8$, $p < 0.001$<br>Group: $F_{(4, 32)} = 0.264$, $p = 0.899$<br>Time x Group: $F_{(56, 434)} = 2.32$, $p < 0.001$<br>**Multiple comparisons**<br>Sham vs 6-OHDA [IC Vehicle]: p = 0.739<br>Sham vs 6-OHDA [IC Secretome]: p = 0.287<br>Sham vs 6-OHDA [IV Vehicle]: p = 0.400<br>Sham vs 6-OHDA [IV Secretome]: p = 0.983<br>6-OHDA [IC Vehicle] vs 6-OHDA [IC Secretome]: p = 0.927<br>6-OHDA [IV Vehicle] vs 6-OHDA [IV Secretome]: p = 0.730<br>6-OHDA [IC Secretome] vs 6-OHDA [IV Secretome]: p = 0.581	-
**Horizontal spontaneous activity**<br>*Nr of squares traveled (1min)*	**After Lesion:** $\chi^2 = 23.93$, $p < 0.001$<br>**After treatments**<br>**1 week:** $\chi^2 = 1.92$, $p = 0.7512$<br>**4 weeks:** $\chi^2 = 8.14$, $p = 0.087$<br>**7 weeks:** $\chi^2 = 9.12$, $p = 0.058$	-
**Gait quality**	**After Lesion:** $\chi^2 = 7.91$, $p = 0.095$<br>**After treatments**<br>**1 week:** $\chi^2 = 6.71$, $p = 0.152$<br>**4 weeks:** $\chi^2 = 8.36$, $p = 0.079$<br>**7 weeks:** $\chi^2 = 12.00$, $p = 0.017$	
**Hindlimb tonus**<br>*Left paw*	**After Lesion:** $\chi^2 = 6.79$, $p = 0.148$<br>**After treatments**<br>**1 week:** $\chi^2 = 13.00$, $p = 0.011$<br>**4 weeks:** $\chi^2 = 14.28$, $p = 0.007$<br>**7 weeks:** $\chi^2 = 13.54$, $p = 0.0089$	

Table S3. Statistical report for the in vivo study.

References

1. Reinhardt, P.; Glatza, M.; Hemmer, K.; Tsytsyura, Y.; Thiel, C.S.; Höing, S.; Moritz, S.; Parga, J.A.; Wagner, L.; Bruder, J.M.; et al. Derivation and Expansion Using Only Small Molecules of Human Neural Progenitors for Neurodegenerative Disease Modeling. *PLoS ONE* **2013**, *8*, e59252, doi:10.1371/journal.pone.0059252.
2. Smits, L.M.; Reinhardt, L.; Reinhardt, P.; Glatza, M.; Monzel, A.S.; Stanslowsky, N.; Rosato-Siri, M.D.; Zanon, A.; Antony, P.M.; Bellmann, J.; et al. Modeling Parkinson's Disease in Midbrain-like Organoids. *npj Parkinson's Disease* **2019**, *5*, 5, doi:10.1038/s41531-019-0078-4.
3. Monzel, A.S.; Hemmer, K.; Kaoma, T.; Smits, L.M.; Bolognin, S.; Lucarelli, P.; Rosety, I.; Zagare, A.; Antony, P.; Nickels, S.L.; et al. Machine Learning-Assisted Neurotoxicity Prediction in Human Midbrain Organoids. *Parkinsonism & Related Disorders* **2020**, *75*, 105–109, doi:10.1016/j.parkreldis.2020.05.011.
